# Supplementary material for: HeteroTCR: A heterogeneous graph neural network-based method for predicting peptide-TCR interaction
Source: Commun Biol. 2024 Jun 4;7:684. doi: 10.1038/s42003-024-06380-6 (PMC11150398; doi:10.1038/s42003-024-06380-6)
Supplement: Supplementary file 2 — Supplementary Information [file 42003_2024_6380_MOESM2_ESM.pdf]

Supplementary Tables

Supplementary Table 1. Details of comparison with HeteroTCR and published methods based on pair-based data sets with 5-fold cross-validation

Model	Fold	VDJdb cs.≥0 (9206 pairs)		VDJdb cs.≥1 (630 pairs)		VDJdb cs.≥2 (150 pairs)		VDJdb cs.≥3 (32 pairs)	
		AUC	Mean (std)	AUC	Mean (std)	AUC	Mean (std)	AUC	Mean (std)
NetTCR-1.0	0	0.5776	0.5802 (0.0058)	0.5729	0.5733 (0.0158)	0.5874	0.6146 (0.0380)	0.5312	0.6363 (0.0691)
	1	0.5792		0.5478		0.5823		0.6094	
	2	0.5905		0.5908		0.5912		0.7109	
	3	0.5772		0.5767		0.6592		0.6582	
	4	0.5767		0.5785		0.6527		0.6719	
NetTCR-2.0	0	0.5754	0.5675 (0.0138)	0.5786	0.5908 (0.0279)	0.6071	0.6566 (0.0394)	0.6309	0.6414 (0.0386)
	1	0.5484		0.5517		0.6897		0.6309	
	2	0.5834		0.6268		0.6732		0.5977	
	3	0.5593		0.5947		0.6220		0.6445	
	4	0.5709		0.6020		0.6910		0.7031	
ERGO_LSTM	0	0.5441	0.5695 (0.0297)	0.5637	0.5696 (0.0105)	0.6624	0.6286 (0.0496)	0.4297	0.5867 (0.1951)
	1	0.5945		0.5821		0.6398		0.8398	
	2	0.5746		0.5689		0.5948		0.4766	
	3	0.5339		0.5558		0.5623		0.4336	
	4	0.6005		0.5775		0.6836		0.7539	
ERGO_AE	0	0.6399	0.6327 (0.0090)	0.6086	0.6058 (0.0250)	0.6166	0.6060 (0.0246)	0.5938	0.5930 (0.0326)
	1	0.6173		0.5720		0.6226		0.5430	
	2	0.6337		0.6247		0.6095		0.6133	
	3	0.6346		0.6332		0.5627		0.5859	
	4	0.6382		0.5903		0.6184		0.6289	
DLpTCR	0	0.6335	0.6237 (0.0110)	0.6121	0.6110 (0.0226)	0.6649	0.6725 (0.0204)	0.7265	0.7539 (0.0814)
	1	0.6236		0.6075		0.6939		0.6523	
	2	0.6297		0.6434		0.6768		0.8555	
	3	0.6052		0.5796		0.6414		0.7188	
	4	0.6263		0.6124		0.6853		0.8164	
HeteroTCR	0	0.6720	0.6658 (0.0045)	0.6588	0.6712 (0.0106)	0.6923	0.7039 (0.0203)	0.6914	0.6992 (0.0175)
	1	0.6626		0.6825		0.7209		0.7266	
	2	0.6693		0.6776		0.7127		0.7031	
	3	0.6618		0.6762		0.6740		0.6797	
	4	0.6634		0.6611		0.7196		0.6953	

The reason for the AUC scores of all models in our dataset not being too high (even not exceeding 0.8) lies in the differences between our experiment's dataset settings and those used in other published approaches. In our setting, the models are trained using a 5-fold cross-validation on the

IEDB dataset and subsequently tested on a separate, independent dataset. This differs from the methodology of previous studies, which entailed training and testing on the same dataset using 5-fold cross-validation, leading to a simple averaging of AUC scores across each validation set to determine the evaluation results. Our setting imposes a stricter criterion on the independent dataset due to potential similarities in spatial distribution within the same database. In contrast, data from different databases exhibit inconsistent spatial distributions. This setting effectively assesses the models' generalization capacity across distinct datasets, revealing its genuine practical value in real-world applications.

Supplementary Table 2. Details of paired t-test P-value between HeteroTCR and any other model based on pair-based data sets with 5-fold cross-validation

	**NetTCR-1.0**	**NetTCR-2.0**	**ERGO_LSTM**	**ERGO_AE**	**DLpTCR**
P-value	4.9×10^{-9}	8.6×10^{-8}	9×10^{-9}	7.9×10^{-7}	1.4×10^{-7}

A paired samples t-test is used to compare the means of two samples when each observation in one sample can be paired with an observation in the other sample. Here, we calculate paired t-test P-value between HeteroTCR and any other model.

Supplementary Table 3. Details of comparison with HeteroTCR and published methods based on four types of data splitting methods with different VDJdb confidence scores

**VDJdb cs.≥0**	**Num**	**Pair-based (9206 pairs)**		**TCR-based (7374 pairs)**		**Antigen-based (1600 pairs)**		**Strict-based (1208 pairs)**	
		**AUC**	**Mean (std)**	**AUC**	**Mean (std)**	**AUC**	**Mean (std)**	**AUC**	**Mean (std)**
NetTCR-1.0	1	0.5336	0.5366 (0.0092)	0.5307	0.5305 (0.0069)	0.4846	0.4984 (0.0084)	0.5097	0.5075 (0.0186)
	2	0.5457		0.5388		0.5008		0.4964	
	3	0.5359		0.5348		0.5076		0.4907	
	4	0.5446		0.5272		0.5003		0.5382	
	5	0.5232		0.5210		0.4988		0.5023	
NetTCR-2.0	1	0.5292	0.5309 (0.0037)	0.5325	0.5469 (0.0222)	0.5031	0.5099 (0.0048)	0.5277	0.5285 (0.0057)
	2	0.5314		0.5304		0.5121		0.5239	
	3	0.5370		0.5294		0.5090		0.5368	
	4	0.5299		0.5697		0.5091		0.5314	
	5	0.5272		0.5727		0.5162		0.5228	
ERGO_LSTM	1	0.5443	0.5527 (0.0392)	0.5099	0.5168 (0.0146)	0.5051	0.5004 (0.0098)	0.4984	0.5207 (0.0213)
	2	0.5948		0.5365		0.4873		0.5165	
	3	0.5923		0.5111		0.5123		0.5392	
	4	0.5215		0.5265		0.4940		0.5463	
	5	0.5107		0.4998		0.5031		0.5033	
ERGO_AE	1	0.5312	0.5427 (0.0268)	0.6137	0.5783 (0.0576)	0.5161	0.5130 (0.0052)	0.5391	0.5138 (0.0203)
	2	0.5237		0.5202		0.5111		0.5281	
	3	0.5445		0.5120		0.5194		0.5141	
	4	0.5884		0.6348		0.5056		0.4912	
	5	0.5259		0.6106		0.5128		0.4965	
DLpTCR	1	0.5858	0.6098 (0.0155)	0.6340	0.6046 (0.0191)	0.5194	0.5167 (0.0101)	0.5127	0.5095 (0.0020)
	2	0.6139		0.5845		0.5292		0.5075	
	3	0.6189		0.5937		0.5201		0.5085	
	4	0.6046		0.5995		0.5126		0.5095	
	5	0.6257		0.6115		0.5020		0.5095	
HeteroTCR	1	0.7029	0.7027 (0.0039)	0.7177	0.7154 (0.0016)	0.7024	0.7004 (0.0060)	0.6611	0.6535 (0.0052)
	2	0.6999		0.7148		0.6955		0.6514	
	3	0.7038		0.7133		0.7098		0.6496	
	4	0.6983		0.7159		0.6988		0.6566	
	5	0.7084		0.7153		0.6956		0.6487	

VDJdb cs.≥1	Num	Pair-based (630 pairs)		TCR-based (416 pairs)		Antigen-based (170 pairs)		Strict-based (108 pairs)	
		AUC	Mean (std)	AUC	Mean (std)	AUC	Mean (std)	AUC	Mean (std)
NetTCR-1.0	1	0.5324	0.5368 (0.0099)	0.5251	0.5436 (0.0183)	0.5684	0.5167 (0.0557)	0.4580	0.4655 (0.0210)
	2	0.5499		0.5736		0.5601		0.4359	
	3	0.5267		0.5453		0.4394		0.4772	
	4	0.5446		0.5393		0.5370		0.4647	
	5	0.5306		0.5349		0.4785		0.4919	
NetTCR-2.0	1	0.5533	0.5546 (0.0105)	0.5559	0.5860 (0.0420)	0.4844	0.5141 (0.0236)	0.5478	0.4999 (0.0444)
	2	0.5440		0.5552		0.5109		0.4506	
	3	0.5514		0.5581		0.5498		0.4544	
	4	0.5722		0.6149		0.5075		0.5249	
	5	0.5520		0.6459		0.5179		0.5219	
ERGO_LSTM	1	0.5641	0.5500 (0.0285)	0.5129	0.5264 (0.0410)	0.4997	0.4950 (0.0072)	0.4583	0.4870 (0.0281)
	2	0.5829		0.5865		0.4839		0.5155	
	3	0.5597		0.5172		0.5026		0.4972	
	4	0.5334		0.5402		0.4958		0.5081	
	5	0.5099		0.4750		0.4931		0.4559	
ERGO_AE	1	0.5155	0.5525 (0.0286)	0.5668	0.5455 (0.0538)	0.5724	0.5450 (0.0337)	0.4767	0.4375 (0.0458)
	2	0.5349		0.5206		0.5525		0.4242	
	3	0.5902		0.4642		0.5145		0.4774	
	4	0.5649		0.5758		0.5055		0.3664	
	5	0.5568		0.6002		0.5802		0.4429	
DLpTCR	1	0.5774	0.6010 (0.0195)	0.5958	0.5875 (0.0119)	0.5799	0.5737 (0.0155)	0.4933	0.4439 (0.0372)
	2	0.6046		0.5836		0.5648		0.4209	
	3	0.6025		0.6016		0.5599		0.4636	
	4	0.5906		0.5706		0.5979		0.4446	
	5	0.6301		0.5859		0.5658		0.3972	
HeteroTCR	1	0.6714	0.6720 (0.0054)	0.7079	0.7039 (0.0067)	0.6912	0.6899 (0.0086)	0.6536	0.6134 (0.0283)
	2	0.6648		0.7066		0.6792		0.5989	
	3	0.6720		0.6946		0.6974		0.6232	
	4	0.6719		0.7110		0.6987		0.6137	
	5	0.6800		0.6996		0.6829		0.5777	

VDJdb cs.≥2	Num	Pair-based (150 pairs)		TCR-based (82 pairs)		Antigen-based (26 pairs)		Strict-based (16 pairs)	
		AUC	Mean (std)	AUC	Mean (std)	AUC	Mean (std)	AUC	Mean (std)
NetTCR-1.0	1	0.5665	0.5756 (0.0114)	0.5845	0.6194 (0.0279)	0.3669	0.4154 (0.0807)	0.5156	0.4359 (0.1089)
	2	0.5698		0.6517		0.4497		0.5000	
	3	0.5668		0.6098		0.3876		0.2500	
	4	0.5920		0.6068		0.5385		0.4844	
	5	0.5829		0.6440		0.3343		0.4297	
NetTCR-2.0	1	0.6107	0.5977 (0.0306)	0.6618	0.7110 (0.0819)	0.3462	0.3337 (0.0385)	0.4922	0.3922 (0.0736)
	2	0.5947		0.6621		0.3728		0.4219	
	3	0.5609		0.6318		0.3639		0.3984	
	4	0.6416		0.8001		0.2899		0.2969	
	5	0.5808		0.7992		0.2959		0.3516	
ERGO_LSTM	1	0.5915	0.5694 (0.0692)	0.4848	0.5205 (0.0759)	0.2840	0.3148 (0.0269)	0.1719	0.2438 (0.1063)
	2	0.6277		0.6092		0.3373		0.3750	
	3	0.6336		0.4402		0.3432		0.3125	
	4	0.5156		0.5937		0.3195		0.1094	
	5	0.4786		0.4747		0.2899		0.2500	
ERGO_AE	1	0.5613	0.6096 (0.0474)	0.6344	0.6128 (0.1071)	0.3403	0.3343 (0.0514)	0.2031	0.1969 (0.0237)
	2	0.5641		0.5562		0.2604		0.1875	
	3	0.6670		0.4640		0.4024		0.2344	
	4	0.6453		0.6645		0.3195		0.1875	
	5	0.6105		0.7448		0.3491		0.1719	
DLpTCR	1	0.5925	0.6503 (0.0500)	0.7002	0.6998 (0.0267)	0.3254	0.4355 (0.0854)	0.1094	0.1625 (0.0650)
	2	0.6014		0.6556		0.4911		0.0938	
	3	0.6923		0.7127		0.5444		0.2031	
	4	0.6686		0.7264		0.4260		0.1563	
	5	0.6967		0.7043		0.3905		0.2500	
HeteroTCR	1	0.6907	0.6866 (0.0081)	0.7412	0.7505 (0.0101)	0.6509	0.6627 (0.0641)	0.9062	0.8969 (0.0237)
	2	0.6768		0.7573		0.6154		0.8594	
	3	0.6868		0.7394		0.6391		0.9062	
	4	0.6811		0.7519		0.7751		0.9219	
	5	0.6976		0.7626		0.6331		0.8906	

VDJdb cs.≥3	Num	Pair-based (32 pairs)		TCR-based (6 pairs)		Antigen-based (8 pairs)		Strict-based (0 pairs)	
		AUC	Mean (std)	AUC	Mean (std)	AUC	Mean (std)	AUC	Mean (std)
NetTCR-1.0	1	0.4844	0.4930 (0.0452)	0.5000	0.5333 (0.0843)	0.4375	0.4250 (0.1300)		
	2	0.5723		0.5556		0.5000			
	3	0.4785		0.6667		0.2812			
	4	0.4688		0.5000		0.5938			
	5	0.4609		0.4444		0.3125			
NetTCR-2.0	1	0.5059	0.4941 (0.0370)	0.6111	0.6778 (0.1542)	0.5000	0.4000 (0.0948)		
	2	0.4629		0.6111		0.3125			
	3	0.5449		0.5000		0.5000			
	4	0.5039		0.7778		0.3750			
	5	0.4531		0.8889		0.3125			
ERGO_LSTM	1	0.4141	0.4742 (0.0641)	0.2222	0.3333 (0.1111)	0.4375	0.4500 (0.0685)		
	2	0.5625		0.4444		0.5625			
	3	0.5195		0.2222		0.4375			
	4	0.4258		0.4444		0.3750			
	5	0.4492		0.3333		0.4375			
ERGO_AE	1	0.5273	0.5281 (0.0738)	0.5000	0.4333 (0.0994)	0.3125	0.4250 (0.1027)		
	2	0.4609		0.3333		0.3750			
	3	0.6523		0.4444		0.5625			
	4	0.5117		0.5556		0.3750			
	5	0.4883		0.3333		0.5000			
DLpTCR	1	0.6367	0.7336 (0.0717)	0.8889	0.7778 (0.2606)	0.6875	0.7125 (0.0948)		
	2	0.7617		0.4444		0.8125			
	3	0.6914		0.5556		0.7500			
	4	0.8242		1.0000		0.5625			
	5	0.7539		1.0000		0.7500			
HeteroTCR	1	0.6953	0.6648 (0.0245)	0.6667	0.6667 (0.0786)	0.6875	0.5500 (0.1118)		
	2	0.6758		0.6667		0.6250			
	3	0.6719		0.5556		0.4375			
	4	0.6328		0.6667		0.5625			
	5	0.6484		0.7778		0.4375			

Supplementary Table 4. Details of paired t-test P-value between HeteroTCR and any other model based on four types of data splitting methods with different VDJdb confidence scores

VDJdb cs.⩾0	NetTCR-1.0	NetTCR-2.0	ERGO_LSTM	ERGO_AE	DLpTCR
P-value	2.22×10^{-16}	2.22×10^{-16}	2.1×10^{-14}	5.1×10^{-14}	1×10^{-12}

VDJdb cs.⩾1	NetTCR-1.0	NetTCR-2.0	ERGO_LSTM	ERGO_AE	DLpTCR
P-value	6.7×10^{-14}	5.5×10^{-12}	1.4×10^{-12}	1.8×10^{-12}	1.1×10^{-11}

VDJdb cs.⩾2	NetTCR-1.0	NetTCR-2.0	ERGO_LSTM	ERGO_AE	DLpTCR
P-value	7.4×10^{-7}	2.3×10^{-5}	8×10^{-7}	1.7×10^{-5}	8.9×10^{-4}

VDJdb cs.⩾3	NetTCR-1.0	NetTCR-2.0	ERGO_LSTM	ERGO_AE	DLpTCR
P-value	6.2×10^{-5}	0.003	1.7×10^{-5}	4.5×10^{-4}	0.99

A paired samples t-test is used to compare the means of two samples when each observation in one sample can be paired with an observation in the other sample. Here, we calculate paired t-test P-value between HeteroTCR and any other model.

Supplementary Table 5. Details of comparison with HeteroTCR and the baseline model based on four types of data splitting methods with VDJdb confidence score greater than or equal to 0

VDJdb cs.≥0	Fold	Pair-based (9206 pairs)		TCR-based (7374 pairs)		Antigen-based (1600 pairs)		Strict-based (1208 pairs)	
		AUC	Mean (std)	AUC	Mean (std)	AUC	Mean (std)	AUC	Mean (std)
Remove Heterogeneous GNN module	0	0.5493	0.5077 (0.0238)	0.5623	0.5139 (0.0300)	0.5054	0.5028 (0.0089)	0.4997	0.5037 (0.0113)
	1	0.5053		0.5090		0.5050		0.4981	
	2	0.4944		0.5115		0.4883		0.5198	
	3	0.4916		0.5073		0.5125		0.5101	
	4	0.4978		0.4794		0.5030		0.4909	
HeteroTCR	0	0.7029	0.7027 (0.0039)	0.7177	0.7154 (0.0016)	0.7024	0.7004 (0.0060)	0.6611	0.6535 (0.0052)
	1	0.6999		0.7148		0.6955		0.6514	
	2	0.7038		0.7133		0.7098		0.6496	
	3	0.6983		0.7159		0.6988		0.6566	
	4	0.7084		0.7153		0.6956		0.6487	

Supplementary Table 6. Details of t-test P-value between HeteroTCR and the baseline model based on four types of data splitting methods with VDJdb confidence score greater than or equal to 0

VDJdb cs.≥0	Pair-based	TCR-based	Antigen-based	Strict-based
P-value	3.8×10^{-5}	1.1×10^{-4}	1.3×10^{-9}	3.6×10^{-7}

Supplementary Table 7. Details of model's sensitivity to the parameter K

K	Num	Pair-based (9206 pairs)		TCR-based (7374 pairs)		Antigen-based (1600 pairs)		Strict-based (1208 pairs)	
		AUC	Mean (std)	AUC	Mean (std)	AUC	Mean (std)	AUC	Mean (std)
1	1	0.6684	0.6686 (0.0035)	0.6697	0.6737 (0.0071)	0.6400	0.6499 (0.0094)	0.6277	0.6216 (0.0056)
	2	0.6696		0.6766		0.6485		0.6240	
	3	0.6627		0.6784		0.6571		0.6245	
	4	0.6709		0.6633		0.6617		0.6137	
	5	0.6712		0.6807		0.6422		0.6180	
2	1	0.6842	0.6895 (0.0030)	0.7018	0.6989 (0.0037)	0.6770	0.6726 (0.0052)	0.6285	0.6324 (0.0057)
	2	0.6908		0.6946		0.6782		0.6394	
	3	0.6917		0.6950		0.6723		0.6261	
	4	0.6911		0.7017		0.6652		0.6374	
	5	0.6896		0.7014		0.6705		0.6308	
3	1	0.7029	0.7027 (0.0039)	0.7177	0.7154 (0.0016)	0.7024	0.7004 (0.0060)	0.6611	0.6535 (0.0052)
	2	0.6999		0.7148		0.6955		0.6514	
	3	0.7038		0.7133		0.7098		0.6496	
	4	0.6983		0.7159		0.6988		0.6566	
	5	0.7084		0.7153		0.6956		0.6487	
4	1	0.7171	0.7157 (0.0011)	0.7149	0.7220 (0.0053)	0.7331	0.7306 (0.0037)	0.6689	0.6661 (0.0054)
	2	0.7165		0.7223		0.7333		0.6737	
	3	0.7154		0.7277		0.7244		0.6631	
	4	0.7154		0.7189		0.7322		0.6595	
	5	0.7142		0.7263		0.7301		0.6653	
5	1	0.7093	0.7084 (0.0052)	0.7078	0.7122 (0.0065)	0.7114	0.7217 (0.0058)	0.6590	0.6663 (0.0054)
	2	0.7034		0.7166		0.7225		0.6676	
	3	0.7028		0.7090		0.7253		0.6740	
	4	0.7143		0.7214		0.7248		0.6662	
	5	0.7123		0.7061		0.7244		0.6648	
6	1	0.7005	0.7047 (0.0028)	0.7013	0.7055 (0.0037)	0.7298	0.7251 (0.0046)	0.6715	0.6668 (0.0041)
	2	0.7050		0.7099		0.7238		0.6692	
	3	0.7046		0.7064		0.7248		0.6664	
	4	0.7084		0.7078		0.7287		0.6607	
	5	0.7051		0.7019		0.7182		0.6660	

Supplementary Table 8. Details of the degree of aggregation of the colored points (per-peptide with ≥ 15 cognate TCRs) is assessed by computing the central position of the point set and the average distance of each point to the center

Peptide Name	Without Heterogeneous GNN Module					With Heterogeneous GNN Module				
	Num 1	Num 2	Num 3	Num 4	Num 5	Num 1	Num 2	Num 3	Num 4	Num 5
NLVPMVATV	0.3203	0.2225	0.3500	0.3091	0.2652	0.1985	0.1881	0.1985	0.1979	0.1958
	0.2934 (0.0500)					0.1957 (0.0044)				
KAFSPEVIPMF	0.5210	0.3760	0.4250	0.5599	0.5721	0.4718	0.6117	0.4816	0.5267	0.4842
	0.4908 (0.0863)					0.5152 (0.0579)				
GTSGSPIINR	0.5082	0.5107	0.4556	0.5329	0.5013	0.3216	0.3774	0.3402	0.3879	0.3462
	0.5018 (0.0284)					0.3547 (0.0274)				
GILGFVFTL	0.4619	0.4948	0.4672	0.6824	0.6155	0.5918	0.5724	0.5333	0.6063	0.5600
	0.5444 (0.0992)					0.5728 (0.0283)				
FPRPWLHGL	0.4613	0.4978	0.3821	0.5260	0.5975	0.2129	0.1799	0.2167	0.2483	0.1989
	0.4929 (0.0796)					0.2114 (0.0252)				
KRWIILGLNK	0.5679	0.5340	0.5018	0.5476	0.5708	0.2995	0.2996	0.2926	0.3547	0.3020
	0.5444 (0.0282)					0.3097 (0.0254)				
FLKEKGGL	0.5984	0.5268	0.6076	0.5090	0.6519	0.3280	0.2965	0.3265	0.3802	0.3118
	0.5787 (0.0594)					0.3286 (0.0315)				
GLCTLVAML	0.1459	0.0850	0.0746	0.0743	0.0691	0.0653	0.0662	0.0635	0.0690	0.0661
	0.0898 (0.0319)					0.0660 (0.0020)				
GTSGSPIVNR	0.3410	0.3193	0.2898	0.3384	0.3197	0.2224	0.2572	0.2298	0.2562	0.2355
	0.3217 (0.0205)					0.2402 (0.0157)				
KLVALGINAV	0.5465	0.5281	0.4806	0.4799	0.4959	0.2855	0.2512	0.2919	0.2804	0.2847
	0.5062 (0.0298)					0.2788 (0.0159)				
ELAGIGILTV	0.3696	0.3964	0.3525	0.3911	0.4032	0.3875	0.3385	0.3501	0.4198	0.3794
	0.3826 (0.0210)					0.3751 (0.0322)				
TPQDLNTML	0.7037	0.4707	0.7320	0.4544	0.5264	0.4038	0.4739	0.3905	0.4480	0.3862
	0.5775 (0.1313)					0.4205 (0.0386)				

Supplementary Table 9. Details of the per-peptide (≥ 15 samples) AUC for the models with and without Heterogeneous GNN module which five repeated trained on IEDB and tested on VDJdb confidence score greater than or equal to 1 based on pair-based setting

Peptide Name	Without Heterogeneous GNN Module					With Heterogeneous GNN Module				
	Num 1	Num 2	Num 3	Num 4	Num 5	Num 1	Num 2	Num 3	Num 4	Num 5
NLVPMVATV	0.3480	0.3519	0.3634	0.4051	0.2870	0.6798	0.6860	0.6481	0.6782	0.6798
	0.3511 (0.0424)					0.6744 (0.0150)				
KAFSPEVIPMF	0.3781	0.3970	0.3459	0.4802	0.3762	0.5992	0.5803	0.6522	0.5860	0.6484
	0.3955 (0.0508)					0.6132 (0.0346)				
GTSGSPIINR	0.8367	0.8163	0.9235	0.8776	0.8929	0.8520	0.7704	0.8469	0.8520	0.8367
	0.8694 (0.0431)					0.8316 (0.0348)				
GILGFVFTL	0.3580	0.5309	0.4938	0.5926	0.4198	0.6543	0.6790	0.7160	0.7037	0.6914
	0.4790 (0.0921)					0.6889 (0.0237)				
FPRPWLHGL	0.7812	0.3438	0.7031	0.7969	0.6094	0.7500	0.6875	0.7031	0.7969	0.7188
	0.6469 (0.1850)					0.7313 (0.0434)				
KRWIILGLNK	0.5306	0.5747	0.5039	0.4694	0.5872	0.5128	0.4777	0.5616	0.5515	0.5431
	0.5332 (0.0489)					0.5293 (0.0341)				
FLKEKGGL	0.5000	0.3438	0.3906	0.5156	0.4375	0.9062	0.7812	0.7969	0.9062	0.9219
	0.4375 (0.0724)					0.8625 (0.0676)				
GLCTLVAML	0.7424	0.7812	0.7424	0.7479	0.7008	0.7867	0.7701	0.7950	0.7950	0.8006
	0.7429 (0.0286)					0.7895 (0.0119)				
GTSGSPIVNR	0.8061	0.7449	0.8061	0.8112	0.8214	0.9388	0.9184	0.9235	0.9541	0.9694
	0.7979 (0.0303)					0.9408 (0.0212)				
KLVALGINAV	0.7188	0.6562	0.7031	0.7656	0.7031	0.7969	0.8438	0.8281	0.8438	0.8125
	0.7094 (0.0392)					0.8250 (0.0204)				
ELAGIGILTV	0.4306	0.3958	0.4792	0.3819	0.3472	0.5694	0.5625	0.5139	0.5833	0.5278
	0.4069 (0.0503)					0.5514 (0.0293)				
TPQDLNTML	0.6875	0.7656	0.6406	0.7812	0.7344	0.5625	0.5312	0.5781	0.5625	0.5625
	0.7219 (0.0578)					0.5594 (0.0171)				

Supplementary Table 10. Details of HeteroTCR AUC across different number of subsampled peptides based on strict-based data sets. The models are five repeated trained on IEDB and tested on VDJdb confidence score greater than or equal to 0

Number of subsampled peptides	Without Heterogeneous GNN Module				
	Num 1	Num 2	Num 3	Num 4	Num 5
100	0.5049	0.5051	0.5056	0.5071	0.5035
	0.5052 (0.0013)				
200	0.5355	0.5368	0.5364	0.5348	0.5398
	0.5367 (0.0019)				
300	0.5846	0.5993	0.6077	0.6057	0.5893
	0.5973 (0.0101)				
400	0.6040	0.6141	0.6077	0.6090	0.6267
	0.6123 (0.0088)				
500	0.6445	0.6472	0.6417	0.6554	0.6469
	0.6471 (0.0051)				
559	0.6611	0.6514	0.6496	0.6566	0.6487
	0.6535 (0.0052)				

Supplementary Figures

Supplementary Figure 1: Performance comparison with HeteroTCR and published methods based on four types of data splitting methods with VDJdb confidence score greater than or equal to 1.

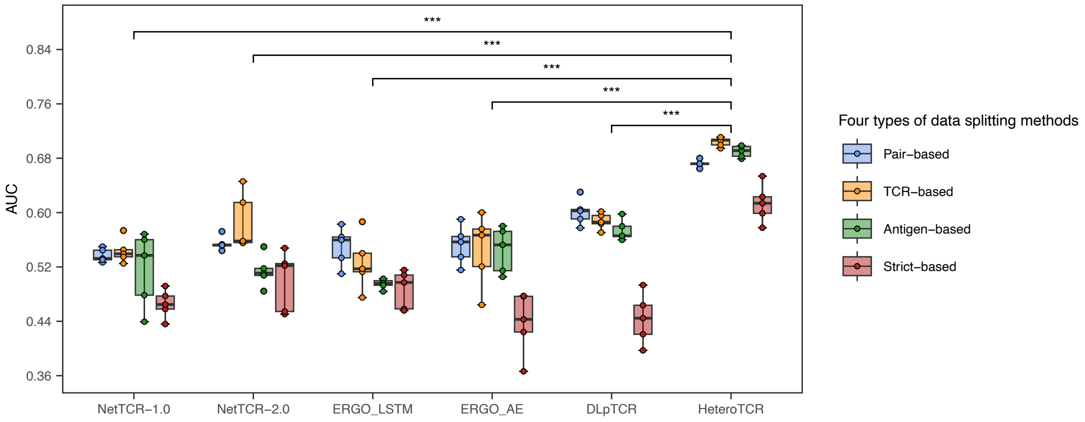

The amount of VDJdb is 630, 416, 170, and 108 for pair-based data sets, TCR-based data sets, antigen-based data sets, and strict-based data sets, respectively. All boxplots: The center line of each boxplot marks the sample median, the colored points scattered along each boxplot represent all the actual data points, and the box extends from the lower to upper quartile. Each boxplot represents the results of n=5 independent experiments. Paired t-test P-value between HeteroTCR and any other model is < 0.00001.

Supplementary Figure 2: Performance comparison with HeteroTCR and published methods based on four types of data splitting methods with VDJdb confidence score greater than or equal to 2.

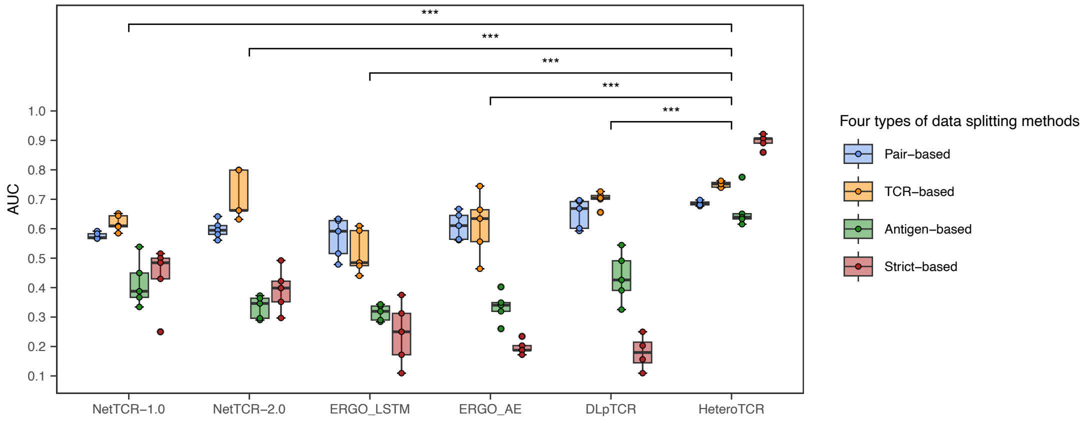

Performance on VDJdb with the confidence score of 2. The amount of VDJdb is 150, 82, 26, and 16 for pair-based data sets, TCR-based data sets, antigen-based data sets, and strict-based data sets, respectively. All boxplots: The center line of each boxplot marks the sample median, the colored points scattered along each boxplot represent all the actual data points, and the box extends from the lower to upper quartile. Each boxplot represents the results of n=5 independent experiments. Paired t-test P-value between HeteroTCR and any other model is < 0.00001.

Supplementary Figure 3: Performance comparison with HeteroTCR and published methods based on four types of data splitting methods with VDJdb confidence score greater than or equal to 3.

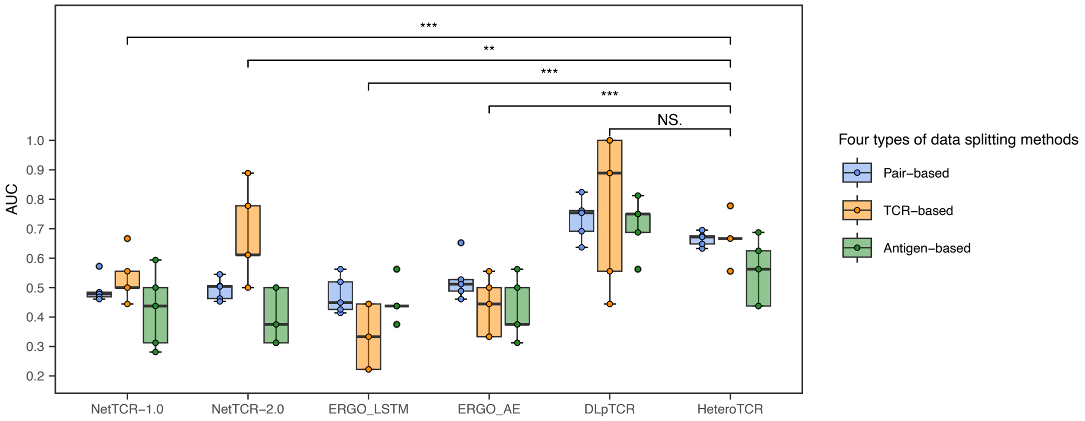

The amount of VDJdb is 32, 6, 8, and 0 for pair-based data sets, TCR-based data sets, antigen-based data sets, and strict-based data sets, respectively. The amount of data in VDJdb with the confidence score greater than or equal to 3 is too small and lacks statistical significance, so we will not consider it. All boxplots: The center line of each boxplot marks the sample median, the colored points scattered along each boxplot represent all the actual data points, and the box extends from the lower to upper quartile. Each boxplot represents the results of n=5 independent experiments. Paired t-test P-value between HeteroTCR and any other model is represented.
