# Supplementary material for: HeteroTCR: A heterogeneous graph neural network-based method for predicting peptide-TCR interaction
Source: Commun Biol. 2024 Jun 4;7:684. doi: 10.1038/s42003-024-06380-6 (PMC11150398; doi:10.1038/s42003-024-06380-6)
Supplement: Supplementary file 3 — Reporting Summary [file 42003_2024_6380_MOESM3_ESM.pdf]

nature portfolio

Corresponding author(s): Xun Lan

Last updated by author(s): May 6, 2024

# Reporting Summary

Nature Portfolio wishes to improve the reproducibility of the work that we publish. This form provides structure for consistency and transparency in reporting. For further information on Nature Portfolio policies, see our Editorial Policies and the Editorial Policy Checklist.

## Statistics

For all statistical analyses, confirm that the following items are present in the figure legend, table legend, main text, or Methods section.

| n/a | Confirmed | |
|---|---|---|
| ☐ | ☒ | The exact sample size (*n*) for each experimental group/condition, given as a discrete number and unit of measurement |
| ☐ | ☒ | A statement on whether measurements were taken from distinct samples or whether the same sample was measured repeatedly |
| ☐ | ☒ | The statistical test(s) used AND whether they are one- or two-sided<br>*Only common tests should be described solely by name; describe more complex techniques in the Methods section.* |
| ☐ | ☒ | A description of all covariates tested |
| ☐ | ☒ | A description of any assumptions or corrections, such as tests of normality and adjustment for multiple comparisons |
| ☐ | ☒ | A full description of the statistical parameters including central tendency (e.g. means) or other basic estimates (e.g. regression coefficient) AND variation (e.g. standard deviation) or associated estimates of uncertainty (e.g. confidence intervals) |
| ☐ | ☒ | For null hypothesis testing, the test statistic (e.g. *F*, *t*, *r*) with confidence intervals, effect sizes, degrees of freedom and *P* value noted<br>*Give P values as exact values whenever suitable.* |
| ☒ | ☐ | For Bayesian analysis, information on the choice of priors and Markov chain Monte Carlo settings |
| ☐ | ☒ | For hierarchical and complex designs, identification of the appropriate level for tests and full reporting of outcomes |
| ☐ | ☒ | Estimates of effect sizes (e.g. Cohen's *d*, Pearson's *r*), indicating how they were calculated |

*Our web collection on statistics for biologists contains articles on many of the points above.*

## Software and code

Policy information about availability of computer code

| | |
|---|---|
| Data collection | No software was used for data collection. |
| Data analysis | We built and used the model 'HeteroTCR' for the downstream data analysis. The source code developed for this study is available on Github: https://github.com/yuzilan/HeteroTCR. |

For manuscripts utilizing custom algorithms or software that are central to the research but not yet described in published literature, software must be made available to editors and reviewers. We strongly encourage code deposition in a community repository (e.g. GitHub). See the Nature Portfolio guidelines for submitting code & software for further information.

## Data

Policy information about availability of data

All manuscripts must include a data availability statement. This statement should provide the following information, where applicable:

- Accession codes, unique identifiers, or web links for publicly available datasets
- A description of any restrictions on data availability
- For clinical datasets or third party data, please ensure that the statement adheres to our policy

All datasets used for training and testing HeteroTCR are shared on the github repository (https://github.com/yuzilan/HeteroTCR) and its Zenodo (https://doi.org/10.5281/zenodo.11120879).
The datasets used for training and testing were collected from IEDB (http://www.iedb.org/), VDJdb (https://vdjdb.cdr3.net/) and McPAS-TCR (http://

friedmanlab.weizmann.ac.il/McPAS-TCR/).
The detailed information of the 10x Genomics cohort is available at https://www.10xgenomics.com/resources/datasets. The raw data for single-cell datasets is available at https://zenodo.org/records/6952657.

## Research involving human participants, their data, or biological material

Policy information about studies with human participants or human data. See also policy information about sex, gender (identity/presentation), and sexual orientation and race, ethnicity and racism.

| | |
|---|---|
| Reporting on sex and gender | No human participants. |
| Reporting on race, ethnicity, or other socially relevant groupings | No human participants. |
| Population characteristics | No human participants. |
| Recruitment | No human participants. |
| Ethics oversight | No human participants. |

Note that full information on the approval of the study protocol must also be provided in the manuscript.

# Field-specific reporting

Please select the one below that is the best fit for your research. If you are not sure, read the appropriate sections before making your selection.

☒ Life sciences ☐ Behavioural & social sciences ☐ Ecological, evolutionary & environmental sciences

For a reference copy of the document with all sections, see nature.com/documents/nr-reporting-summary-flat.pdf

# Life sciences study design

All studies must disclose on these points even when the disclosure is negative.

| | |
|---|---|
| Sample size | In this work, we collected data from three well-known databases: IEDB, VDJdb, and McPAS-TCR. To construct reliable datasets, we took four preprocessing steps on the raw data. Therefore, the numbers of peptide-TCR binding pairs in IEDB, VDJdb, and McPAS-TCR are 38174, 8225, and 1785, respectively. |
| Data exclusions | We took four preprocessing steps on the raw data.<br>Step 1. filtering data: (i) The data used in this study were restricted to TCRβ chain sequences due to the scarcity of paired chain data. (ii) Peptide-CDR3β pairs were adopted because the complementarity-determining region 3 (CDR3) is the key determinant of specificity in antigen recognition. (iii) Given that peptide with lengths ranging from 8 to 15 amino acids (AAs) and CDR3β sequences containing 10-20 AAs account for the majority of datasets, we focused on the CDR3β sequences with 10-20 AAs and peptides with 8-15 AAs presented by human MHC class I molecules, as they are likely to represent the most common and biologically relevant cases.<br>Step 2. removing invalid sequences: Since the majority of CDR3β chains begin with the amino acid 'C' and end with 'F', we excluded peptide-CDR3β pairs whose CDR3β sequences do not adhere to this pattern. Furthermore, we eliminated peptide-CDR3β sequences containing lowercase letters or letters that are not amino acids as these likely result from errors during database collection and collation.<br>Step 3. removing redundant sequence pairs: Duplicate sequence pairs may exist in the databases due to the omission of information such as the CDR1 and CDR2 regions and HLA molecules. To address this, we removed redundant data within each database by retaining only unique peptide-CDR3 β sequence pairs.<br>Step 4. screening antigen-specific TCR CDR3s: iSMART is a computational method that performs a specially parameterized pairwise local alignment on TCR CDR3 sequences to group them into antigen-specific clusters with high efficiency. Since TCRs usually share conserved sequence features when recognizing the same pMHC epitope, we employed iSMART as a filter to identify and cluster antigen-specific TCR CDR3s, removing the CDR3s which are not selected in the cluster. |
| Replication | We ran the example python code to confirm our results are reproducible. |
| Randomization | Randomly drawn disjoint training and testing folds were used to evaluate our model.<br>In the Results 2.2 section, we used IEDB dataset as the training dataset with 5-fold cross-validation to generate five optimal parameter models, and evaluated these five models on an independent testing dataset (VDJdb) to get five AUC metrics.<br>In the Results 2.3 section, we trained the model on the data from IEDB dataset, took McPAS-TCR as the validation dataset to select optimal parameter model, and evaluated the model on VDJdb. We repeated this process five times, so we got five optimal parameter models each time and five AUC metrics on the independent testing dataset.<br>In the Results 2.4 section, to avoid being accused of manipulating data using complex filtering steps, we directly used the data of SOTA models to train and evaluate HeteroTCR and compared the results in their papers. |
| Blinding | In this experiment, the investigators understood group allocation and the investigators were blinded to group allocation. |

# Reporting for specific materials, systems and methods

We require information from authors about some types of materials, experimental systems and methods used in many studies. Here, indicate whether each material, system or method listed is relevant to your study. If you are not sure if a list item applies to your research, read the appropriate section before selecting a response.

## Materials & experimental systems

| n/a | Involved in the study | |
|---|---|---|
| ☒ | ☐ | Antibodies |
| ☒ | ☐ | Eukaryotic cell lines |
| ☒ | ☐ | Palaeontology and archaeology |
| ☒ | ☐ | Animals and other organisms |
| ☒ | ☐ | Clinical data |
| ☒ | ☐ | Dual use research of concern |
| ☒ | ☐ | Plants |

## Methods

| n/a | Involved in the study | |
|---|---|---|
| ☒ | ☐ | ChIP-seq |
| ☒ | ☐ | Flow cytometry |
| ☒ | ☐ | MRI-based neuroimaging |

## Plants

| | |
|---|---|
| Seed stocks | No plants. |
| Novel plant genotypes | No plants. |
| Authentication | No plants. |
